# Ultrasound Shear Elastography With Expanded Bandwidth (USEWEB): A Novel Method for 2D Shear Phase Velocity Imaging of Soft Tissues

**DOI:** 10.1109/TMI.2024.3352097

**Published:** 2024-05-02

**Authors:** Piotr Kijanka, Matthew W. Urban

**Affiliations:** Department of Robotics and Mechatronics, AGH University of Krakow, 30-059 Kraków, Poland.; Department of Radiology, and the Department of Physiology and Biomedical Engineering, Mayo Clinic, Rochester, MN 55905 USA

**Keywords:** Shear wave elastography (SWE), ultrasound, acoustic radiation force (ARF), dispersion, phantom, elastic, viscoelastic, inclusion, Stockwell transform

## Abstract

Ultrasound shear wave elastography (SWE) is a noninvasive approach for evaluating mechanical properties of soft tissues. In SWE either group velocity measured in the time-domain or phase velocity measured in the frequency-domain can be reported. Frequency-domain methods have the advantage over time-domain methods in providing a response for a specific frequency, while time-domain methods average the wave velocity over the entire frequency band. Current frequency-domain approaches struggle to reconstruct SWE images over full frequency bandwidth. This is especially important in the case of viscoelastic tissues, where tissue viscoelasticity is often studied by analyzing the shear wave phase velocity dispersion. For characterizing cancerous lesions, it has been shown that considerable biases can occur with group velocity-based measurements. However, using phase velocities at higher frequencies can provide more accurate evaluations. In this paper, we propose a new method called Ultrasound Shear Elastography with Expanded Bandwidth (USEWEB) used for two-dimensional (2D) shear wave phase velocity imaging. We tested the USEWEB method on data from homogeneous tissue-mimicking liver fibrosis phantoms, custom-made viscoelastic phantom measurements, phantoms with cylindrical inclusions experiments, and *in vivo* renal transplants scanned with a clinical scanner. We compared results from the USEWEB method with a Local Phase Velocity Imaging (LPVI) approach over a wide frequency range, i.e., up to 200–2000 Hz. Tests carried out revealed that the USEWEB approach provides 2D phase velocity images with a coefficient of variation below 5% over a wider frequency band for smaller processing window size in comparison to LPVI, especially in viscoelastic materials. In addition, USEWEB can produce correct phase velocity images for much higher frequencies, up to 1800 Hz, compared to LPVI, which can be used to characterize viscoelastic materials and elastic inclusions.

## I. INTRODUCTION

Changes in mechanical properties can occur due to soft tissue pathology. Over the past three decades, several methods have been developed to measure the mechanical properties of soft tissues. Shear wave elastography (SWE), which includes various implementations such as supersonic shear imaging and comb-push ultrasound shear elastography, is one such class of methods [1], [2], [3]. These techniques use acoustic radiation force (ARF) to generate shear waves, while ultrasound techniques are employed to measure the resulting shear wave motion.

SWE techniques have been employed to image various tissues, such as the liver, breast, thyroid, skeletal muscle, kidney, and prostate [3]. Among these, the primary use of SWE techniques has been for staging liver fibrosis, which has shown good success [4]. SWE has also been extensively employed for tumor characterization in various organs such as the breast, thyroid, and prostate [5], [6], [7]. In the context of cancer imaging applications, it has been observed that inclusions found in breast, thyroid, and liver tissues are typically stiffer than the surrounding normal tissue, and malignant lesions are typically stiffer than benign lesions [5], [7], [8], [9], [10], [11]. In clinically implemented SWE techniques, certain assumptions are made regarding the medium being imaged. These assumptions include the medium being elastic, locally homogeneous, isotropic, and incompressible. When imaging inhomogeneities of finite size, it is important to consider resolution as a significant factor.

There are several techniques for reconstructing the mechanical properties in SWE, specifically the shear wave velocity. The majority of these methods rely on time-domain data and involve the local measurement of shear wave time-of-flight [12], [13]. Frequency-domain approaches can also be used for estimating shear wave velocity from the data. Phase gradient or Fourier transform (FT) methods have been commonly employed for this purpose [14], [15]. These methods are utilized to determine the phase velocity dispersion due to material viscoelasticity and geometry [16], [17], [18], [19]. However, most of these measurement techniques have limited region-of-interest (ROI).

Recently, the Local Phase Velocity based Imaging (LPVI) approach was proposed as an alternative-technique to measure tissue elasticity [20], [21], [22], [23], [24]. The LPVI approach is fast and works very well for elastic homogeneous and heterogeneous soft materials, but struggles with viscoelastic media and image reconstruction for higher frequencies. This problem can be reduced by using a narrow-band filter in the wavenumber domain (k-filter) centered about the nominal wavenumber of the shear wave-mode [22]. However, it can be difficult to find *a priori* the nominal wavenumber distribution for *in vivo* studies.

We propose a new technique called Ultrasound Shear Elastography with Expanded Bandwidth (USEWEB) to generate images of phase velocity in soft tissues across a wider range of frequencies. The unique approach used in this method uses a generalized Stockwell transform along with short space slant wavenumber-frequency analysis. The technique we propose combines the benefits of the LPVI and GST-SFK methods that have been previously developed and evaluated [20], [21], [22], [23], [25], [26]. By utilizing these methods, we aim to enhance the advantages of previous techniques while also expanding the range of frequencies that can be imaged.

The main novelty of this work is the proposed new USEWEB approach, which offers the capability to construct 2D images of viscoelastic phantoms/tissues across an extended frequency range with a significantly reduced coefficient of variation (<5%), which, to the best of our knowledge, is not possible with any other SWE method. This extended frequency range opens the door for new differentiation capabilities for viscoelastic tissues [26]. Additionally, with the improved robustness images with higher resolution can be obtained and provide more accurate characterization of heterogeneous inclusions [27].

## II. METHODS

### A. Ultrasound Shear Elastography With Expanded Bandwidth (USEWEB)

**Fig. 1.**
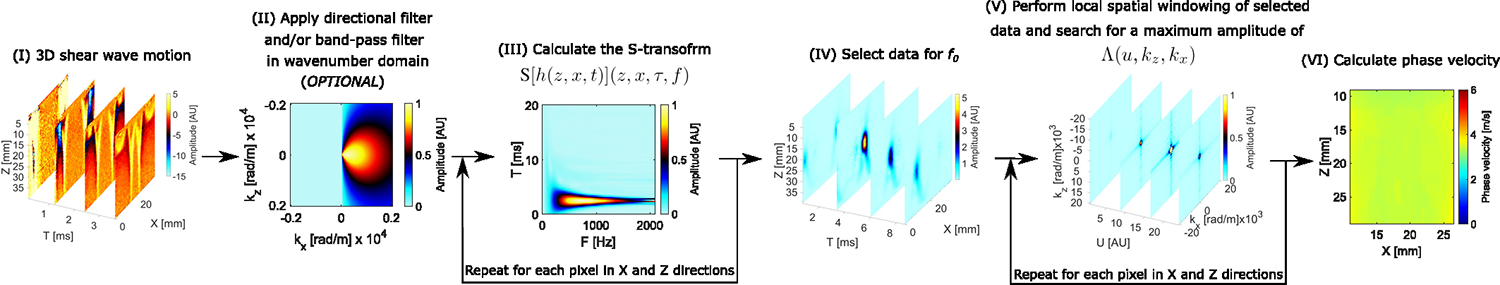
Flowchart of the proposed USEWEB approach. The principal steps of the USEWEB can be summarized as follows: (I) Acquire a 3D shear wave velocity data. (II) Apply directional filter to the data and/or band-pass filter in a wavenumber domain (k-filter) if necessary. (III) Transform 3D spatiotemporal data into 4D time-frequency-space-space domain (t,f,z,x) using Eq. (1). (IV) Choose the spectrum at a particular frequency $f_{0}$. (V) Search for a maximum amplitude of $\Lambda \left(k_{z},k_{x},u\right)$ over all steering group velocities, u, to obtain the frequency-wavenumber pairs K ($k_{z},k_{x},f_{0}$), using Eq. (6). (VI) Calculate spatial distribution of the phase velocity of the shear wave motion for the specified frequency.

The newly proposed USEWEB approach operates in a time-frequency-wavenumber domain, providing additional information about shear wave modes and wavenumber distribution compared to the time-space and frequency-wavenumber domains. The proposed approach employs a generalized S-transform to produce a time-frequency decomposition of the spatiotemporal particle motion signal v(z,x,t) with a frequency-dependent Gaussian window used for spectral localization. To summarize, the steps of the USEWEB approach (illustrated in a flowchart in Fig. 1) used for shear wave phase velocity imaging can be described as follows.

First, we acquire three-dimensional (3D) shear wave particle velocity motion data. Next, we process the data by applying a directional filter or a band-pass filter in a wavenumber domain as needed. Then, we decompose the 3D spatiotemporal particle motion signal v(z,x,t) into the four-dimensional (4D) time-frequency-space-space domain (t,f,z,x) using a generalized S-transform, given as [25]

(1) $$S[v(\tau )](z,\;x,\;\tau ,\;f)=\int_{-\infty }^{+\infty }\;v(z,\;x,\;t)W(\tau -t,\;f)e^{-i2\pi ft}dt,$$

where, f is a frequency and τ is a parameter which controls the position of the Gaussian window on the time vector, t.W is the scaled Gaussian window given as

(2) $$W(\tau -t,f)=\frac{\left|f\right|}{\sqrt{2\pi \beta }}e^{-\frac{f^{2}{\left(\tau -t\right)}^{2}}{2\beta }},$$

where, β is a scaling factor. The scaling factor β determines the width of the window used in the S-transform, thereby influencing the time-frequency resolution of the analysis. Specifically, a narrower window in the time domain corresponds to a wider window in the frequency domain, leading to reduced frequency resolution. Conversely, larger β values result in a wider Gaussian window in the time domain, which enhances frequency resolution.

Then, a collection of complex-valued functions in time and distance can be generated for a chosen frequency $f_{0}$ or range of frequencies, which can be written as

(3) $$H(z,\;x,\;\tau )=S[h(z,\;x,\;t)]\left(z,\;x,\;\tau ,\;f_{0}\right).$$

Next, the H(z,x,τ) function is divided into small segments across spatial dimensions by multiplying it with a window function. This function has a non-zero value only within a small region in space and remains constant over the entire frequency domain. For example, a two-dimensional (2D) cosine-tapered window (i.e., Tukey window) can be used [20], [21]. When the window slides along the spatial dimensions, windowed $H(\overset{‾}{z},\;\overset{‾}{x},\;\tau )$ functions are generated.

The complex-valued slant-phase function (SF) can be obtained by taking slant slices of the $H(\overset{‾}{z},\;\overset{‾}{x},\;\tau )$ function at a selected frequency $f_{0}$ or range of frequencies, steering group velocity $u=\overset{‾}{x}/\tau$, and constant time, which can be written in a form

(4) $$SF(\overset{‾}{z},\;\overset{‾}{x})=H\left(\overset{‾}{z},\overset{‾}{x},\frac{\overset{‾}{x}}{u_{m}}=\tau \right)\;\text{for}\;u_{m}=\frac{{\overset{‾}{x}}_{m}}{t_{m}\;-\;m\Delta t}.$$

The SF function is computed for a series of steering group velocity values $u_{m}$, for a maximum lateral distance, ${\overset{‾}{x}}_{m}$, and a maximum time, $t_{m}$, of the recorded shear wave motion data. The time sampling rate is denoted by Δt. Next, the amplitude of the SF function is calculated as

(5) $$\Lambda \left(k_{z},k_{x},u,f_{0}\right)=\left|\int_{-\infty }^{+\infty }\;\;SF(\overset{‾}{z},\overset{‾}{x})e^{-2i\pi \left(k_{z}\overset{‾}{z}+k_{x}\overset{‾}{x}\right)}dxdz\right|,$$

which is a 4D spectral amplitude distribution with the coordinates of the steering group velocity, frequency, and wavenumbers in z and x directions. The spectral amplitude peaks of the SF function represent the distribution of wavenumbers of elastic waves traveling away from the source. To derive the local wavenumber, the maximum amplitude of $\Lambda \left(k_{z},k_{x},u,f_{0}\right)$ across all steering group velocities is used, which can be considered as

(6) $$K\left(k_{z},k_{x},f_{0}\right)=\underset{u}{max}\;[\Lambda \left(k_{z},k_{x},u,f_{0}\right)].$$

The peaks of $K\left(k_{z},k_{x},f_{0}\right)$ for an impulsive ARF push are related to the phase velocities of different wave propagation modes [28].

In the USEWEB approach, the last step involves determining the spatial distribution of the phase velocity of shear wave motion at the frequency $f_{0}$ or range of frequencies. Phase velocity is computed from finding the peaks in the $K\left(k_{z},k_{x},f_{0}\right)$ distribution as

(7) $$c_{ph(z,x)}=\frac{2\pi f_{0}}{\left|k\right|},$$

where |k| is a wavenumber magnitude described as

(8) $$|k|=\sqrt{k_{z}^{2}+k_{x}^{2}},$$

and $k_{z}$ and $k_{x}$ arguments are found using

(9) $$[k_{z},k_{x}]=\underset{\left(k_{z},k_{x}\right)}{arg\;max}[K\left(k_{z},k_{x},f_{0}\right)].$$

The entire field-of-view (FOV) of the phase shear wave velocity image is reconstructed using Eq. (7). It should be emphasized that the frequency $f_{0}$ can be either a single frequency value or a frequency band, $f_{band}$, centered around $f_{0}\left(f_{0}-f_{b},\ldots ,f_{0},\ldots ,f_{0}+f_{b}\right)$.

We implemented the aforementioned procedure using MATLAB R2022a (Mathworks, Natick, MA) to demonstrate and evaluate its underlying principle and basic performance.

### B. Local Phase Velocity Imaging (LPVI)

The LPVI method was originally proposed in [20] and further evaluated and tested in [21], [22], and [23]. The main idea behind this approach is, first, to use a one-dimensional FT to transform the spatiotemporal data into a frequency domain. Then, select the spatial spectrum at a particular frequency and perform a short-space 2D-FT on the windowed wave-field regions in the space domains (axial and lateral) to calculate the phase velocity based on the dominant local wavenumber. The LPVI approach described in [21] was used for comparison purposes in this work.

The results for LPVI were presented both without applying the k-filter, as in the USEWEB approach, and with the adoption of this filter. To remove spatial wavelengths representing shear wave velocities outside a predetermined range, a first-order Butterworth bandpass filter is applied to each frame. For the homogeneous phantoms the wavenumber bandwidth is centered around the nominal wavenumber $f_{0}\left(f_{0}-f_{b},\ldots ,f_{0},\ldots ,f_{0}+f_{b}\right)$ of the shear wave-mode and is selected as $V_{GST-SFK}\left(f_{0}\right)\pm 1\;m/s$, where $V_{GST-SFK}\left(f_{0}\right)$ is the phase velocity estimated from the dispersion curve calculated at the focal depth, as described in Section II-C.

### C. One-Dimensional Dispersion Comparison

The following USEWEB and LPVI results for the homogeneous phantoms were compared against the phase velocity dispersion estimates for 2D-FT and the GST-SFK approaches. The 2D-FT and GST-SFK methods were implemented according to [25]. First, the directional filter was used to isolate waves traveling from left-to-right and right-to-left. Then, 2D particle velocity field was averaged along the depth dimension over 1.69 mm, centered at the focal depth. The particle velocity signals were measured in the lateral segment length over 27 mm. Next, the DC component was removed from the waveforms, and dispersion curves were then calculated.

## III. MATERIALS

### A. Liver Fibrosis Tissue Mimicking Homogeneous Phantoms Description

Commercially available liver fibrosis tissue mimicking homogeneous phantoms (Model 039, CIRS Inc., Norfolk, VA, USA, manufactured in 2014) were used in this work to test the USEWEB approach for shear wave phase velocity reconstruction. Phantoms were made with a sound speed of 1540 m/s and ultrasound attenuation of 0.5 dB/cm/MHz. The set of three phantoms with differing Young’s modulus (E) of 10, 25 and 45 kPa was tested in this paper. According to the information provided by manufacturer all phantoms are with a precision of ±4%. A programmable ultrasound research system (V1, Verasonics, Inc., Kirkland, WA, USA) with a 128-element linear array transducer (L7–4, Philips Healthcare, Andover, MA, USA) was used. The push duration was 400 *μs* and the push frequency was 4.09 MHz. Two simultaneous pushes were generated by 44 active elements on the left and right sides of the probe. The focal depth was set to 20 mm. Plane wave compounding with three plane waves at 5 MHz and angles of −4°, 0°, and +4° was used for motion detection. The effective frame rate after compounding was 4.167 kHz, and the spatial sampling was 0.154 mm. The in-phase/quadrature (IQ) data were used to calculate the shear wave particle velocity motion using an auto-correlation algorithm [29].

### B. Tissue-Mimicking Viscoelastic Phantoms Description

Another set of phantoms used in our experiments for the USEWEB approach evaluation were custom-made tissue-mimicking viscoelastic phantoms (CIRS Inc., Norfolk, VA, USA, manufactured in 2017–2018). Three different TM phantoms (designated as A, B, and C) were used, with no reference dispersion curve, similar to those employed in [21], [23], [25], [28], [30], and [31]. Parameters of the viscoelastic standard linear solid (SLS) model for these phantoms were identified by solving a nonlinear least-squares fit problem [32]. The SLS model is composed of spring element, Young’s modulus $E_{1}$, in series with a Kelvin-Voigt element (spring, $E_{2}$, in parallel with a dashpot, η). The results were as follows: $E_{1}=9.61\;kPa,\;E_{2}=15.62\;kPa,\;\eta_{1}=5.28\;Pa\cdot s$ for Phantom A, $E_{1}=18.56\;kPa,\;E_{2}=31.52\;kPa,\;\eta_{1}=7.35\;Pa\cdot s$ for Phantom B, and $E_{1}=12.61\;kPa,\;E_{2}=100\;kPa,\;\eta_{1}=21.45\;Pa\cdot s$ for Phantom C. The same ultrasound research scanner and measurement set-up as for the liver fibrosis phantoms in Section III-A was used for data acquisition.

### C. CIRS Phantom With Inclusions

To evaluate the robustness of the USEWEB approach for shear wave phase velocity imaging in heterogeneous materials, we employed the CIRS elastography phantom with stepped cylindrical inclusions (Model 049A, CIRS Inc., Norfolk, VA, USA, manufactured in 2013). The phantom comprises stepped cylinders of varying sizes and locations, centered around 30 and 60 mm from the phantom surface. The background Young’s modulus is 29 kPa, while the inclusion stiffness values for Type I to IV lesions are 11, 16, 48, and 80 kPa, respectively. Our study focused on testing Type IV lesions with ARF push beams focused at 30 mm, using a push duration of 400 *μ*s and push frequency of 4.09 MHz. The push beam was generated using 32 active elements, positioned 16 elements away from each end of the L7–4 probe, and placed on both sides of the inclusion. Data were processed in the same way as the liver fibrosis and tissue-mimicking viscoelastic phantoms.

### D. In Vivo Renal Transplant Data

The feasibility of the USEWEB method was also tested on data from two *in vivo* renal transplants. Shear wave measurements were performed on human subjects who were scheduled to undergo protocol biopsy. The kidney imaging and measurements were conducted prior to the biopsy, following a protocol approved by the Mayo Clinic Institutional Review Board, and written informed consent was obtained from the participants. The examinations were carried out by an experienced clinical sonographer. The ultrasound probe was positioned to find a longitudinal plane of the kidney, and ROI was placed in the middle of the kidney to make measurements in the renal cortex. The data acquisition was performed using a Logiq E9 ultrasound system equipped with a C1–6-D curved array transducer (General Electric Company, Wauwatosa, WI, USA). The ARF push beams were focused at the edges of the ROI, and directional filtering was applied to the shear wave field to extract the leftward and rightward traveling shear waves. The shear wave motion was measured using ultrasound data with a frame rate of 2.41 kHz.

## IV. RESULTS

### A. Tissue Mimicking Liver Fibrosis Phantoms

**Fig. 2.**
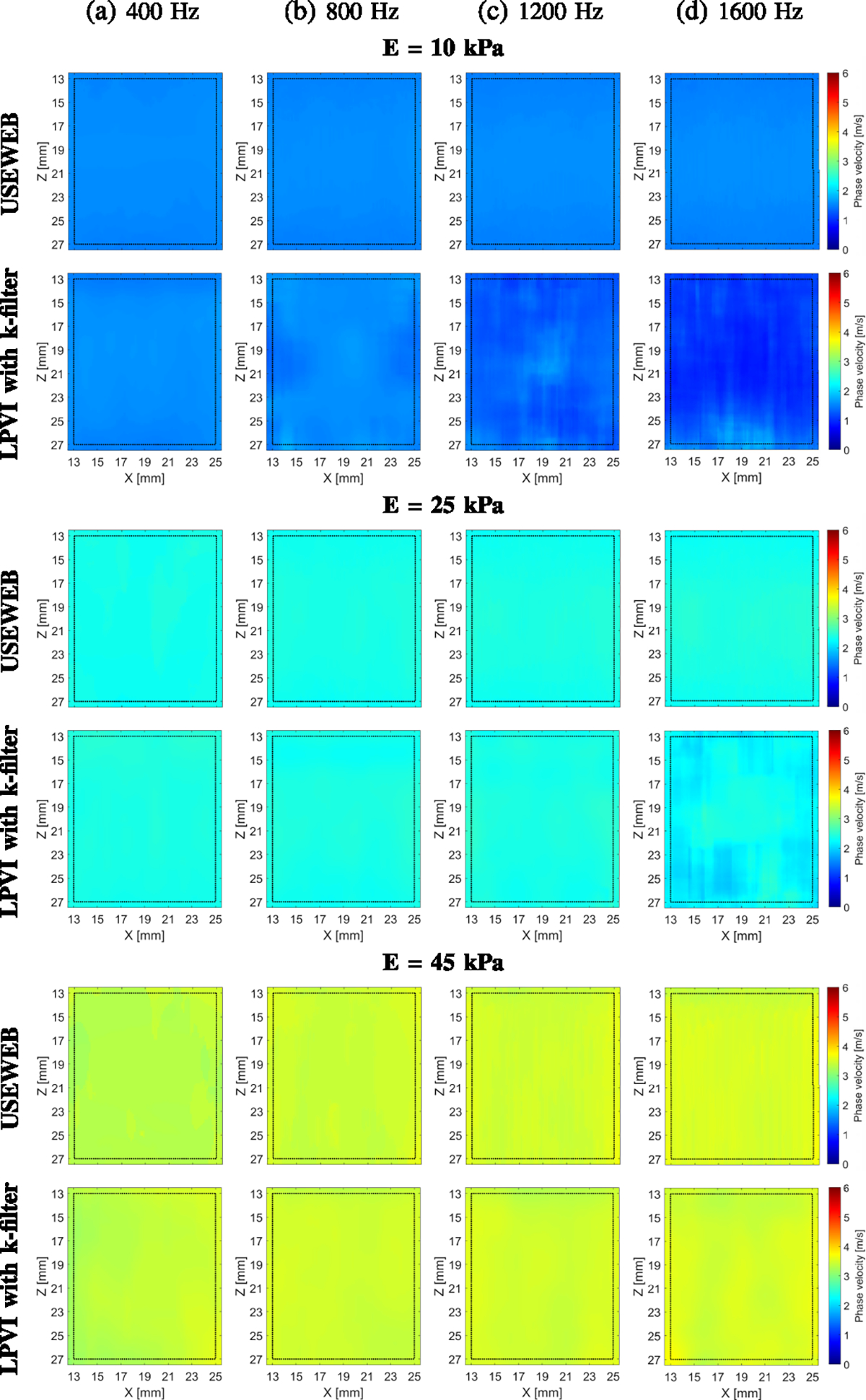
2D shear wave phase velocity images for the liver fibrosis tissue mimicking homogeneous phantoms with Young’s modulus (E) of 10, 25 and 45 kPa, respectively. The images were calculated for a constant spatial window dimension of 4.47 × 4.47 mm. Phase velocity images were calculated based on the USEWEB and LPVI with k-filter approaches for various, selected frequencies (a) 400, (b) 800, (c) 1200, and (d) 1600 Hz, respectively. Dashed lines present a ROI selected for mean and standard deviation values calculations.

In Fig. 2, the final 2D shear wave phase velocity images for homogeneous liver fibrosis tissue mimicking phantoms are shown, reconstructed using the USEWEB and LPVI with k-filter approaches. These images were obtained by averaging reconstructed intermediate maps from two acquisitions, using a spatial window size of 4.47 × 4.47 mm. The figures depict maps for four different frequencies. Because the phantoms in Fig. 2 were expected to exhibit elastic behavior, we did not anticipate any variations in the reconstructed images with varying frequency. When comparing the newly proposed USEWEB approach to the LPVI technique, some differences can be observed. The phase velocity maps reconstructed by USEWEB remain consistent within the selected ROI for all phantoms and frequencies tested. However, the LPVI approach encounters difficulties in reconstructing full ROI images. Specifically, LPVI struggles with frequencies of 800 Hz and above for the softest phantom (E = 10 kPa), and fails at 1600 Hz for a stiffer phantom with a Young’s modulus of 25 kPa. Nonetheless, the LPVI approach successfully provides full reconstructions for all frequencies of the stiffest phantom (E = 45 kPa).

**Fig. 3.**
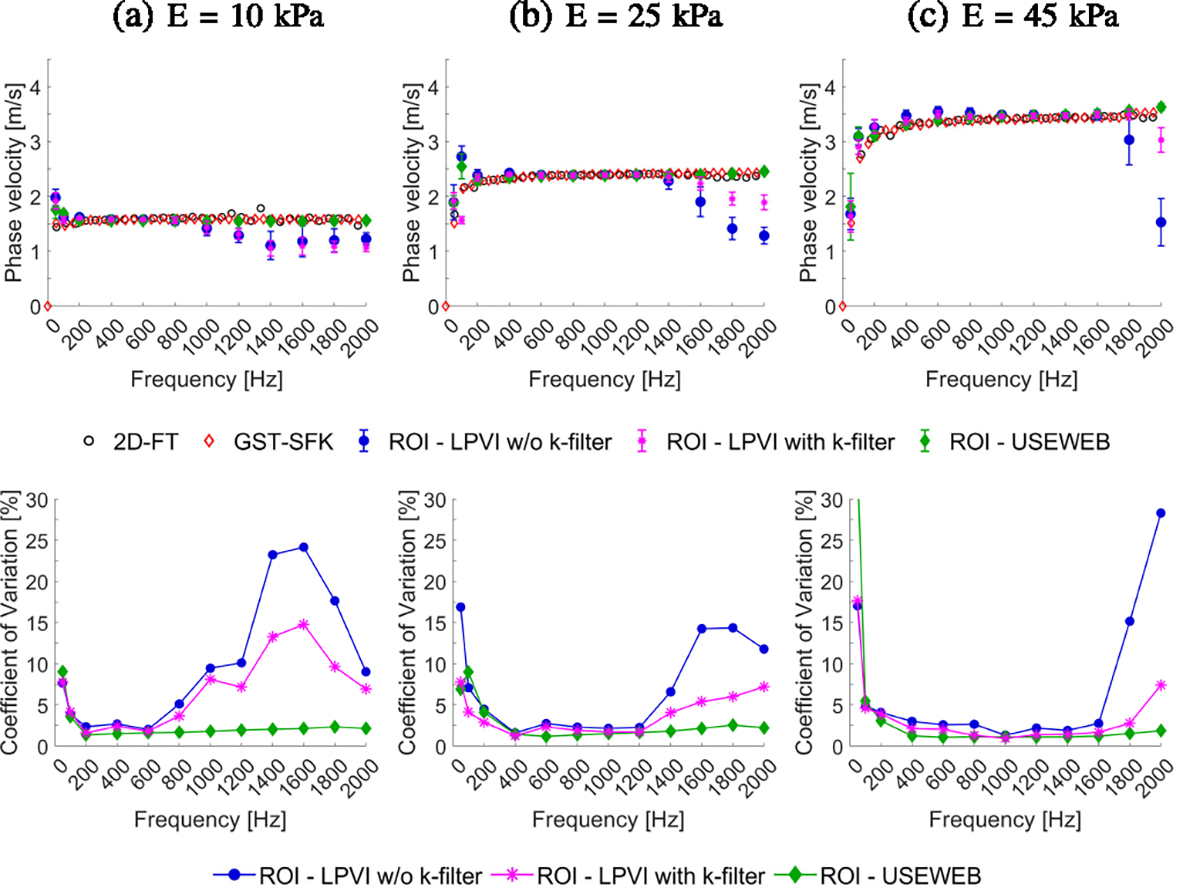
Dispersion phase velocity curves (top row) calculated for the liver fibrosis tissue mimicking homogeneous phantoms using the 2D-FT (black circles), GST-SFK (red diamonds) methods for the focal depth of 20 mm, and mean and standard deviation of the phase velocity for LPVI w/o k-filter (blue circles), LPVI with k-filter (magenta stars) and USEWEB (green diamonds). Corresponding coefficient of variation values, CV = SD/MEAN·100%, for reconstructed images are presented in the bottom row. The mean and standard deviation were calculated for ROIs presented in Fig. 2.

In Fig. 3 top row, dispersion phase velocity curves are presented, which were obtained using both the classical 2D-FT method and the recently proposed GST-SFK approach, at a focal depth of 20 mm. Mean shear wave velocity and standard deviation values were also calculated for each phantom using both the USEWEB and LPVI with and without k-filter approaches, within the ROIs marked in Fig. 2, facilitating a comparison between the two methods. These metrics were computed across various frequencies ranging from 50–2000 Hz for all phantoms. As the frequency increases, the mean phase velocity values calculated for the ROIs using the USEWEB approach show a good correspondence to the dispersion curves calculated at the focal depth for all phantoms, as can be observed in Fig. 3. In contrast, the LPVI approach exhibits deviations from the focal dispersion curves as the frequency increases. These deviations vary depending on the stiffness of the phantom, as shown in Fig. 3. Furthermore, the standard deviation of the mean phase velocity is lower for the USEWEB approach compared to the LPVI technique, particularly at higher frequencies. For phantoms with a range of 10–45 kPa, the USEWEB approach had the highest observed standard deviation of 0.04 m/s at 1800 Hz, 0.09 m/s at 200 Hz, and 0.10 m/s at 200 Hz. In contrast, the LPVI method had the highest standard deviation of 0.28 m/s at 1600 Hz, 0.27 m/s at 1600 Hz, and 0.46 m/s at 1800 Hz, for the same phantoms.

The bottom row of Fig. 3 shows coefficient of variation, defined as $CV=\frac{SD}{MEAN}\cdot 100\%$, calculated for the liver fibrosis tissue mimicking phantoms. The CV coefficient indicates the level of variation relative to the sample mean, with lower values indicating less variation. Comparing the USEWEB and LPVI methods, the CV coefficient remains similar up to 600 Hz for E = 10 kPa, up to 1200 Hz for E = 25 kPa, and up to 1600 Hz for E = 45 kPa. Beyond these frequencies, the CV coefficient for the USEWEB method remains stable, while for the LPVI methods it increases. Both techniques showed increased CV coefficient for frequencies ⩽100 Hz because of the large wavelengths compared to the window size used, i.e., λ⩾18.3,28.9, and 38.7 mm, for E = 10, 25, and 45 kPa, respectively.

**Fig. 4.**
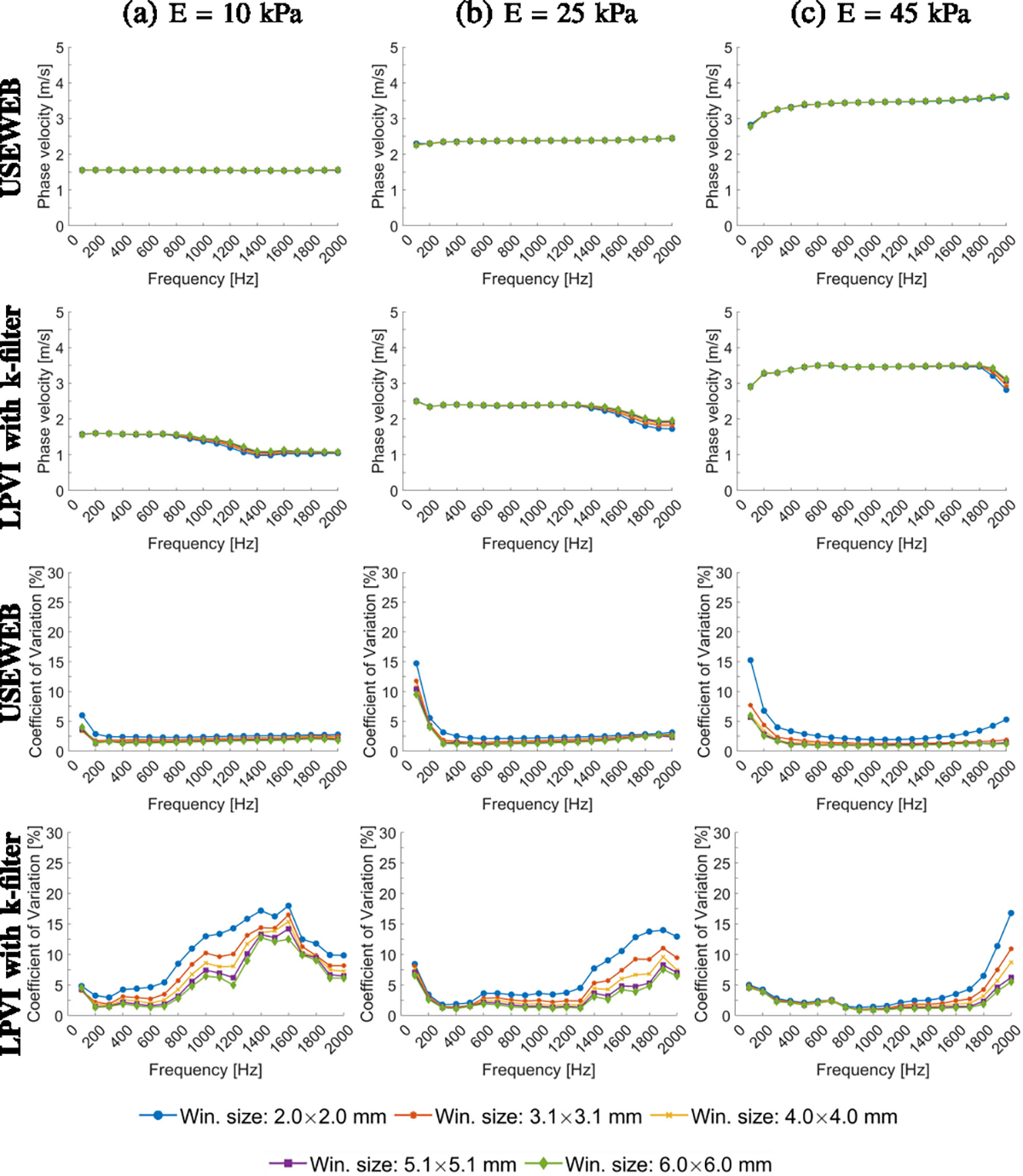
Mean phase velocity calculated for the liver fibrosis tissue mimicking homogeneous phantoms using the USEWEB and LPVI with k-filter methods, for various frequencies and the spatial window dimensions are shown in the first and second rows, respectively. Corresponding coefficient of variation values, CV = SD/MEAN・100%, for reconstructed images are presented in the third and fourth rows. The mean and CV were calculated for ROIs presented in Fig. 2.

Figure 4 presents the mean shear wave velocity and CV calculated within ROIs marked in Fig. 2. These metrics were calculated for various frequencies from 100 to 2000 Hz, the spatial window dimensions varying from 2.0 × 2.0 to 6.0 × 6.0 mm. A larger window size encompasses data from a broader spatial area, resulting in smoother reconstructed images compared to a smaller window size. Calculated mean phase velocity is consistent across all window sizes tested for the given phantoms and USEWEB. Similar behavior can be observed using the LPVI method within the frequency range up to 600 Hz for E = 10 kPa, up to 1200 Hz for E = 25 kPa, and up to 1600 Hz for E = 45 kPa. The CV remains consistent across all phantoms and window sizes greater than 2.0×2.0 mm when using the USEWEB method with a CV of less than 5% for frequencies above 100%. Greater variations in the CV coefficient are apparent with the LPVI technique and varying window sizes.

### B. Tissue Mimicking Viscoelastic Phantoms

**Fig. 5.**
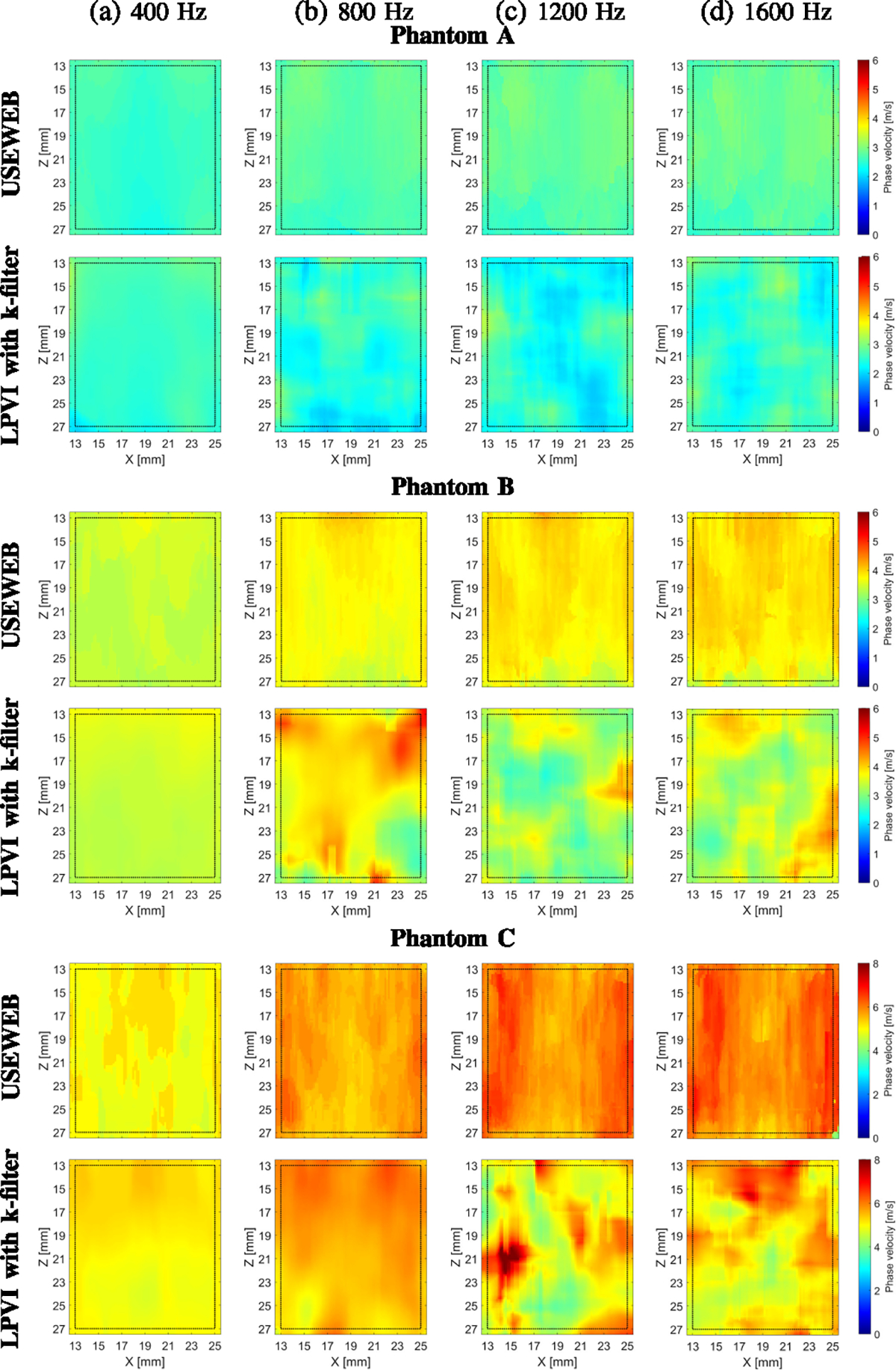
2D shear wave phase velocity images for the tissue mimicking viscoelastic phantoms A-C. The images were calculated for a constant spatial window dimension of 4.47 × 4.47 mm. Phase velocity images were calculated based on the USEWEB and LPVI with k-filter approaches for selected frequencies (a) 400, (b) 800, (c) 1200, and (d) 1600 Hz, respectively. Dashed lines present a ROI selected for mean and standard deviation values calculations.

Figure 5 displays 2D images of shear wave phase velocity for three experimental tissue-mimicking viscoelastic phantoms data. These images were reconstructed for a constant spatial window dimension of 4.47 × 4.47 mm using the USEWEB and LPVI with k-filter methods and depict the results at four different frequencies. Because the phantoms are viscoelastic, it is anticipated that there will be a gradual rise in phase velocity with frequency. Similar to the liver fibrosis phantoms, the USEWEB method produced superior estimates than the LPVI method. However, the advantage of using the USEWEB method is much greater in this case. Across all evaluated frequencies and within the entire ROI, the USEWEB approach yielded smooth phase velocity reconstructions, while the LPVI method often failed after 400 Hz. At higher frequencies, some heterogeneity was observed for phantom B in the USEWEB approach, possibly due to small phantom heterogeneity.

**Fig. 6.**
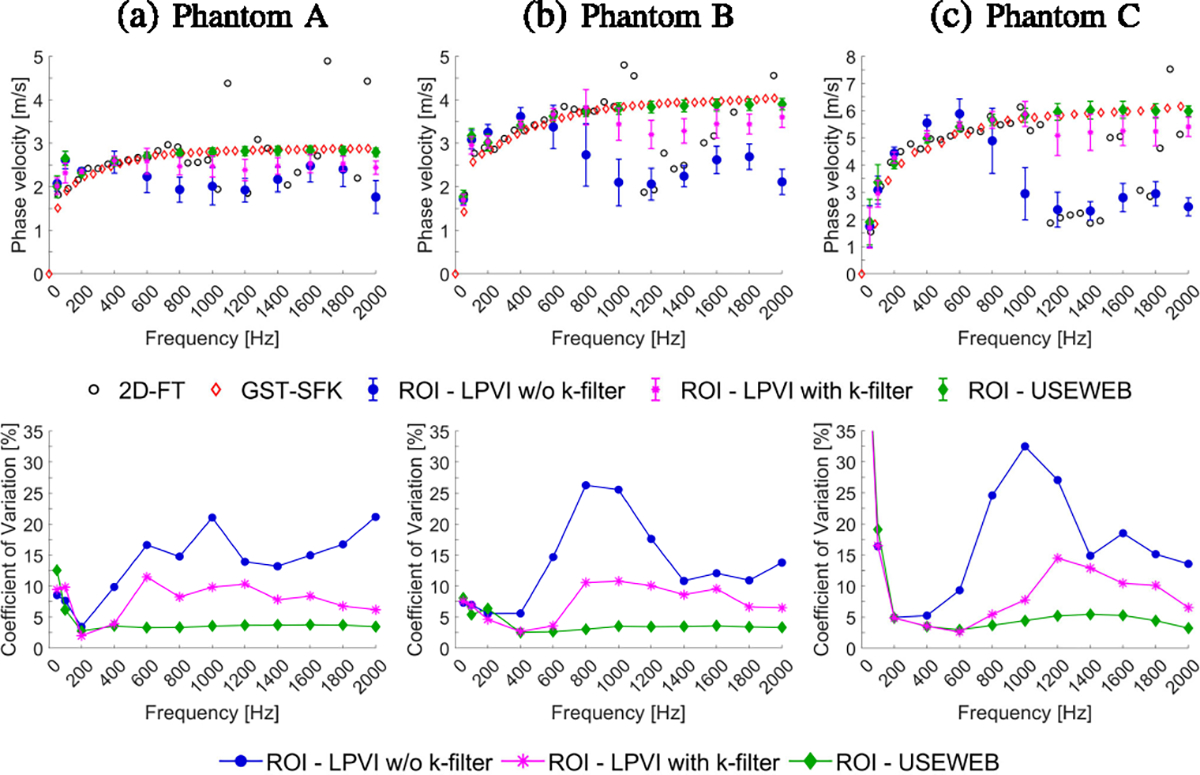
Dispersion phase velocity curves (top row) calculated for the tissue mimicking viscoelastic phantoms A-C using the 2D-FT (black circles), GST-SFK (red diamonds) methods for the focal depth of 20 mm, and mean and standard deviation of the phase velocity for LPVI w/o k-filter (blue circles), LPVI with k-filter (magenta stars) and USEWEB (green diamonds). Corresponding coefficient of variation values, CV = SD/MEAN·100%, for reconstructed images are presented in the bottom row. The mean and standard deviation were calculated for ROIs presented in Fig. 5.

Figure 6 top row displays the phase velocity curves for tissue-mimicking viscoelastic phantoms, calculated using the 2D-FT and GST-SFK methods for the focused excitation push beam depth. Mean phase velocity values for the ROIs, obtained using both USEWEB and LPVI with and without k-filter approaches, are also presented in the figure. The ROIs for each phantom are marked in Fig. 5. Because the classical 2D-FT approach is not effective at higher frequencies in these phantoms, a comparison was made with the more reliable GST-SFK approach, which has previously been examined using numerical and experimental data [25].

The newly proposed USEWEB approach produced phase velocity maps with mean values that closely match the dispersion curves (GST-SFK) calculated at the focal depth, for all phantoms and the entire frequency range considered. Furthermore, the standard deviation within selected ROIs was very low for phantoms A and B. A higher standard deviation was observed for the phantom C, which may be influenced by the increased heterogeneity of the phantom. In contrast, the LPVI approach reconstructed 2D velocity maps with mean phase velocity values that correlate with the dispersion curves at frequencies up to approximately 500 Hz. Additionally, the standard deviation of the phase velocity within ROIs was more than twice as large for the LPVI approach compared to the USEWEB technique for most frequencies and phantoms.

The bottom row of Fig. 6 shows the CV calculated for the tissue mimicking viscoelastic phantoms. It is evident that the USEWEB method exhibited a CV < 5% for all phantoms and frequency bandwidths from 200 Hz to 2000 Hz. The LPVI method with k-filter had comparable CV values (<5%) up to 400 Hz for Phantom A, and up to 600 Hz for Phantoms B and C. However, the CV values increased for higher frequencies. On the other hand, the LPVI method without k-filter exhibited the highest CV values for all phantoms and frequencies examined. Note that for LPVI and USEWEB the spatial resolution depends largely on the spatial wavelengths, which are longer at lower frequencies, which results in larger CV values for frequencies below 200 Hz. This is also the case with 1D methods used for phase velocity dispersion curves calculation such as 2D-FT and GST-SFK.

**Fig. 7.**
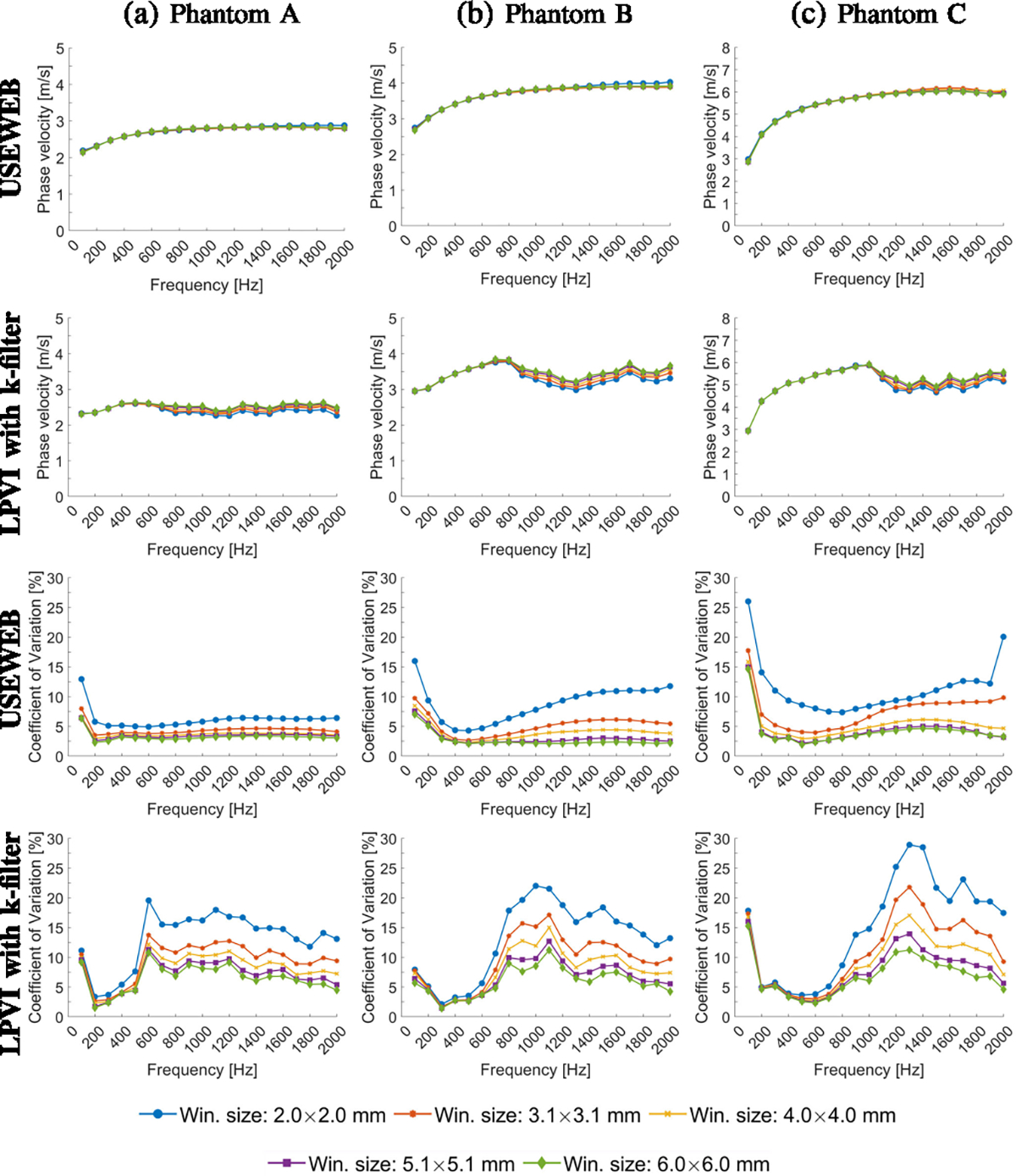
Mean phase velocity calculated for the tissue mimicking viscoelastic phantoms A-C using the USEWEB and LPVI with k-filter methods, for various frequencies and the spatial window dimensions are shown in the first and second rows, respectively. Corresponding coefficient of variation values, CV = SD/MEAN·100%, for reconstructed images are presented in the third and fourth rows. The mean and CV were calculated for ROIs presented in Fig. 5.

Figure 7 in the first and second rows, similarly as in Fig. 4, shows mean phase velocity calculated for the tissue mimicking viscoelastic phantoms A-C using the USEWEB and LPVI with k-filter methods, for various frequencies and the spatial window dimensions. The third and fourth rows of the same figure depict the corresponding CV coefficients. Again, the mean phase velocity calculated for USEWEB is consistent for various window sizes over almost the entire frequency range, exhibiting minimal deviations for the smallest window size evaluated at the highest frequencies. The LPVI method performs similarly up to 600 Hz for Phantom A, 800 Hz for Phantom B, and 1000 Hz for Phantom C. The CV coefficients begin to stabilize when the window size is 4.0 × 4.0 mm or larger using the USEWEB approach. However, the LPVI method exhibits a greater variation in the CV as the window size changes.

### C. CIRS Phantom With Inclusions

**Fig. 8.**
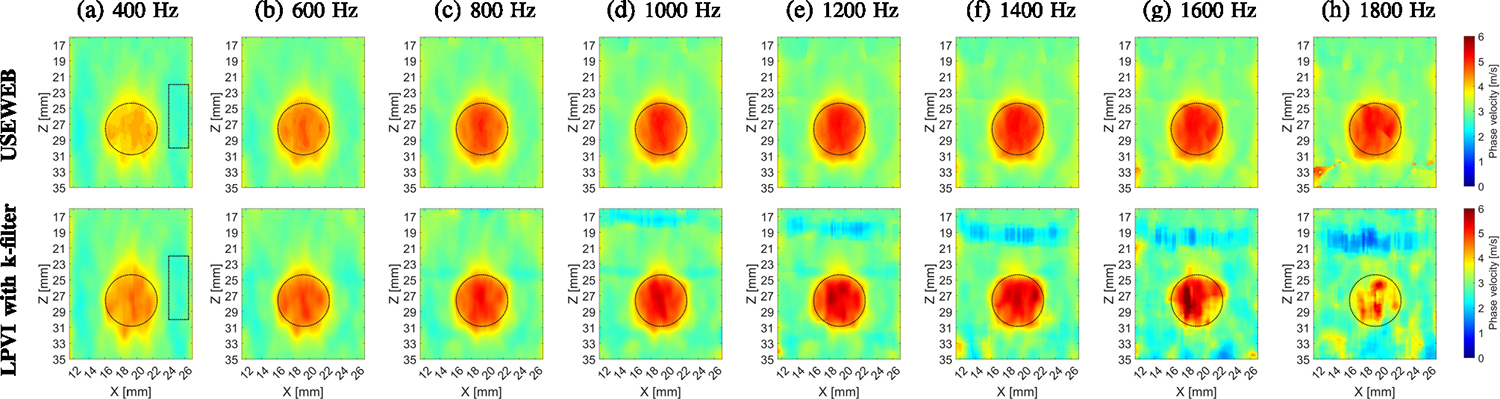
2D shear wave phase velocity images, reconstructed for the inclusion size of 6.49 mm, calculated for a constant spatial window dimension of 2.31 × 2.31 mm and various, selected frequencies from (a) 400 Hz to (h) 1800 Hz, respectively. Presented images are computed for the CIRS phantom with an inclusion Type IV, calculated based on the USEWEB (top row) and LPVI with k-filter (bottom row) approaches. Dashed circular lines present a true inclusion location estimated from B-mode. The dashed rectangular box in (a) represents the area utilized for the contrast-to-noise ratio (CNR) calculations in Fig. 10.

Figure 8 shows the final reconstructed 2D images of the shear wave phase velocity for the inclusion size of 6.49 mm diameter and the USEWEB and LPVI with k-filter methods, respectively. A spatial window dimension of 2.31 × 2.31 mm was kept constant. Frequencies ranging from 400–1800 Hz were selected and used. Presented images were computed for the CIRS phantom with an inclusion Type IV. At the lowest frequency investigated (Fig. 8a), both techniques produced the least robust reconstructed inclusion images. As the selected frequency increases, the image quality of both methods improves in terms of uniformity of the phase velocity and proper reconstruction of the inclusion shape. Additionally, the magnitude of the shear wave velocity increases with frequency, resulting in better contrast in the final images. For higher frequencies, the shape distortions observed at lower frequencies were eliminated, as seen for example in Fig. 8d. Notably, the USEWEB method produced higher quality inclusion images compared to the LPVI method at higher frequencies. USEWEB provided good images for frequencies up to 1800 Hz, while LPVI was limited to 1200 Hz. The quality of the LPVI reconstructions deteriorates from 1400 Hz onwards, as evident in Figs. 8f–8h (bottom row).

**Fig. 9.**
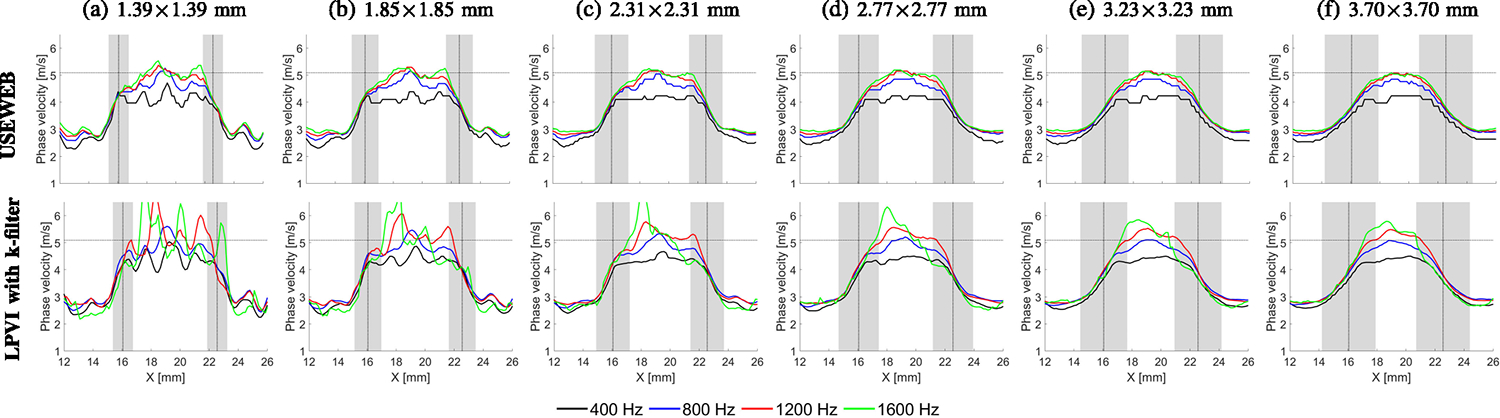
Horizontal cross-section profiles at the central depth of the 6.49 mm inclusion for the CIRS phantom shown in Fig. 8. The results are plotted for various window dimensions, (a)-(f), and selected frequencies of 400, 800, 1200, and 1600 Hz, for the USEWEB (top row) and LPVI with k-filter (bottom row) approaches, respectively. The horizontal, dash-dotted line corresponds to the nominal phase velocity value provided by the manufacturer. The vertical, dotted lines represent a true inclusion location, and the shaded area corresponds to the width of the spatial window used in that location.

Figure 9 illustrates horizontal cross-sectional profiles for the reconstructed inclusion size of 6.49 mm. Inclusion reconstructions were performed using various window dimensions and selected frequencies of 400, 800, 1200, and 1600 Hz. The edge reconstruction can be observed and compared with different combinations of window size and frequency, including results for both the USEWEB and LPVI methods. The vertical dotted black lines in the figure represent the edges of the inclusions estimated based on the B-mode images, while the shaded area corresponds to the width of the spatial window used in that location. The horizontal, dash-dotted line corresponds to the nominal shear wave velocity $\left(V=\sqrt{E/(3\rho )}\right)$ value provided by the manufacturer.

It is apparent that the USEWEB method offers a more reliable quantitative assessment for frequencies above 1200 Hz. In contrast, the LPVI method tends to yield overestimated responses within this frequency range. The ability to obtain accurate values with low variability using smaller spatial window sizes is a key aspect that brings great benefits to potential clinical applications to provide enhanced accuracy with higher spatial resolution.

Using a wider window size resulted in smoother reconstructed profiles for both methods. However, the LPVI method produced more distorted profiles compared to the USEWEB approach. The USEWEB method showed stabilized results for frequencies starting from 1200 Hz and a window size of 2.77 × 2.77 mm (Fig. 9d) or larger, while some variations in the LPVI profiles were still present at the same frequencies. The reconstructed edges for both methods are highly dependent on the spatial window size used. As the window size increases, the sharpness of the inclusion edge decreases, and the transition between background and inclusion materials becomes approximately equal to the window size. This can be observed for the USEWEB approach in Fig. 9 (top row).

**Fig. 10.**
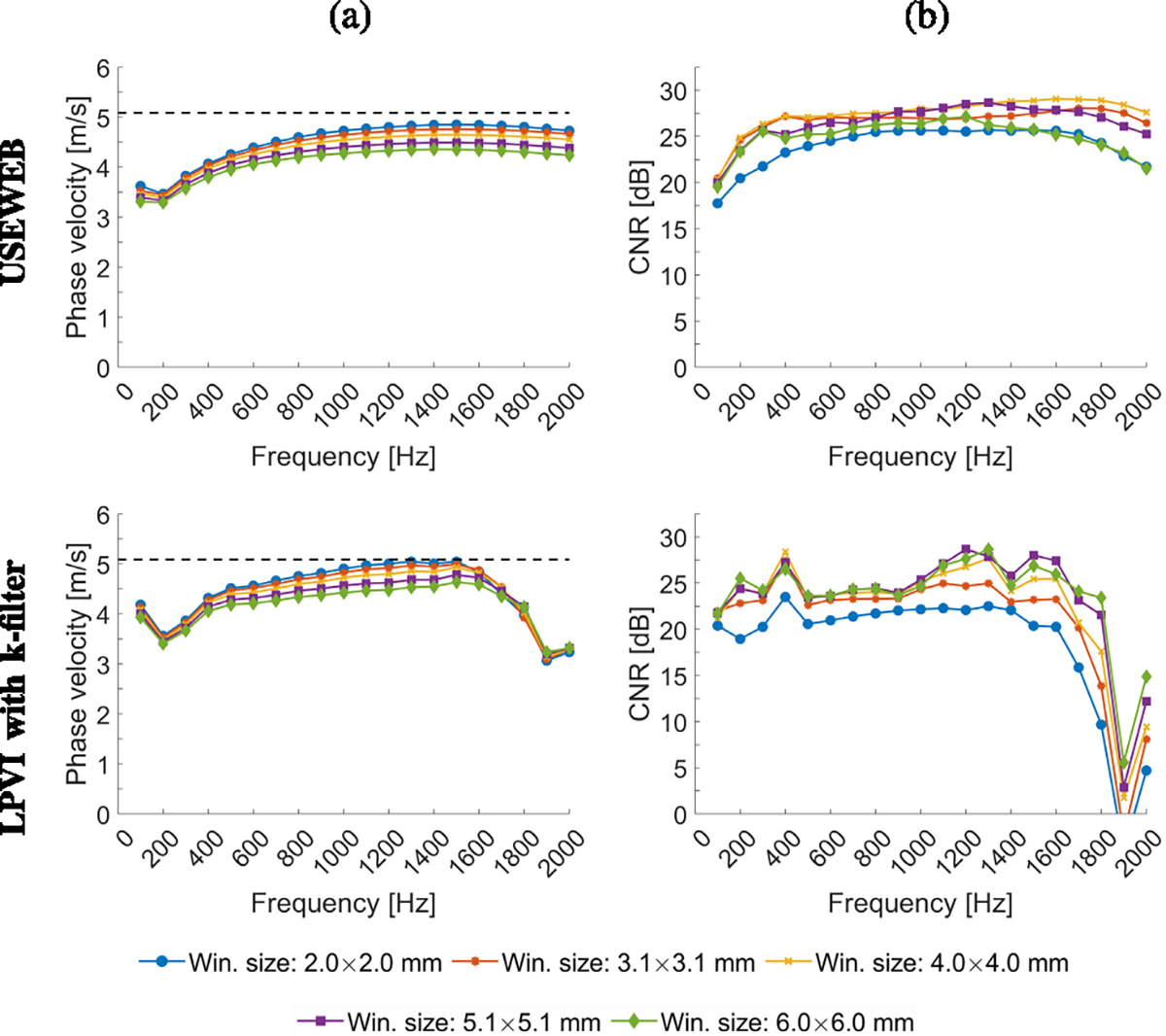
(a) Mean phase velocity calculated for the CIRS phantom with an inclusion Type IV and the size of 6.49 mm using the USEWEB and LPVI with k-filter methods, respectively, for various frequencies and the spatial window dimensions. Corresponding contrast-to-noise ratio (CNR), for reconstructed images are presented in (b). The mean and CNR were calculated for ROIs presented in Fig. 8. The horizontal dashed line in (a) corresponds to the nominal phase velocity value provided by the manufacturer.

Figure 10a shows mean phase velocity computed within an inclusion size of 6.49 mm diameter for various frequencies and the spatial window dimensions, calculated for the USEWEB and LPVI with k-filter methods. The horizontal dashed line corresponds to the nominal phase velocity value provided by the manufacturer. For both methods progressive mean phase velocity increase with increasing frequency was observed up to 1600 Hz for USEWEB, and up to 1400 Hz for LPVI. This behavior is observed for all spatial windows dimensions investigated. Above these frequencies, a slight decrease in the mean phase velocity was present for USEWEB, while for the LPVI method a sudden drop was noticed. The increase in mean phase velocity across the examined frequency range is likely due to changes in wavelength. As frequency increases, shorter wavelengths are present, resulting in a greater velocity. Wavelengths or a larger portion of a wavelength exist within the inclusion. The reduction in phase velocity at the highest frequencies is likely attributable to the heightened sensitivity of the shortest waves to background noise.

In Fig. 10b, the contrast-to-noise ratio (CNR) of the USEWEB and LPVI methods is presented for various spatial window dimensions and various frequencies. The CNR was computed as $\text{CNR}=20\;{log}_{10}\cdot \frac{\left|MEAN_{Inclusion}-MEAN_{Background}\right|}{SD_{Background}}$, where MEAN and SD are the mean standard deviation of shear wave velocity values [20]. It is shown that with increasing the spatial window from 2.0 × 2.0 mm to 6.0 × 6.0 mm the CNR increases about 5 dB. Additionally, the CNR decreases above approximately 1300 Hz for the LPVI technique and 1600 Hz for the USEWEB method, similar to the drop in mean phase velocity values.

**Fig. 11.**
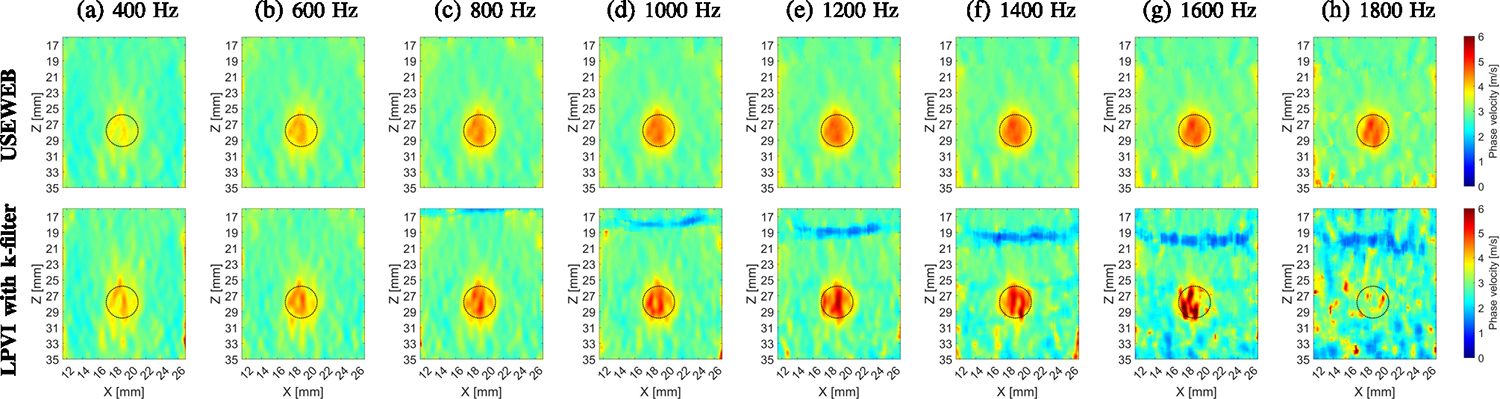
2D shear wave phase velocity images, reconstructed for the inclusion size of 4.05 mm, calculated for a constant spatial window dimension of 1.39 × 1.39 mm and various, selected frequencies from (a) 400 Hz to (h) 1800 Hz, respectively. Presented images are computed for the CIRS phantom with an inclusion Type IV, calculated based on the USEWEB (top row) and LPVI with k-filter (bottom row) approaches. Dashed circular lines present a true inclusion location estimated from B-mode.

Figure 11 shows reconstructed 2D images of the shear wave phase velocity for the inclusion size of 4.05 mm diameter. A spatial window dimension of 1.39 × 1.39 mm was kept constant, and frequencies ranging from 400–1800 Hz were examined. Similar results in Fig. 8 for the USEWEB and LPVI methods were tested and compared.

At 400 Hz and 600 Hz, both methods produced distorted inclusion reconstructions, although the USEWEB method has less noise than the LPVI method. The quality of reconstructed inclusion profiles shows improvement as frequency increases, up to 1200 Hz for the USEWEB method, and up to 1400 Hz for the LPVI method. While the USEWEB method does not show any noticeable improvement nor issues beyond these frequencies, the LPVI method becomes increasingly affected by noise.

### D. In Vivo Renal Transplant Data

**Fig. 12.**
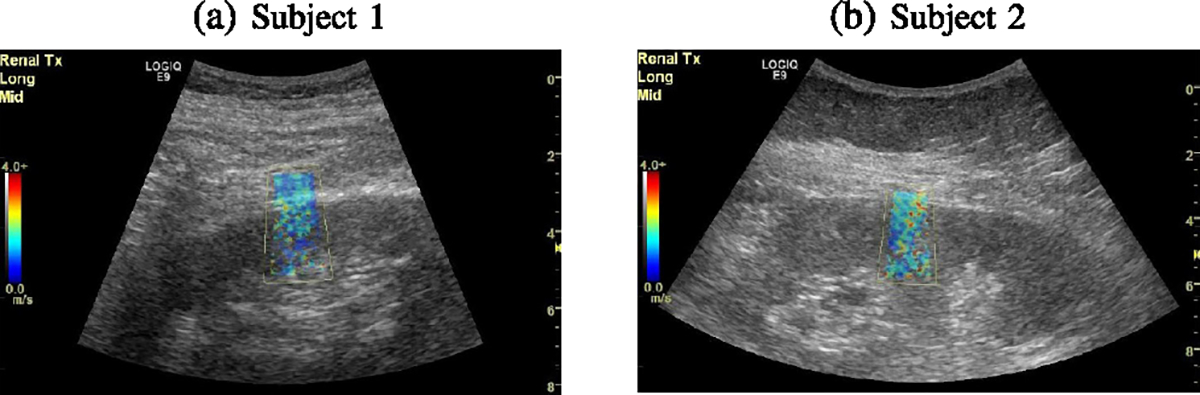
B-mode images for the *in vivo* renal transplant data, for (a) Subject 1 and (b) Subject 2.

**Fig. 13.**
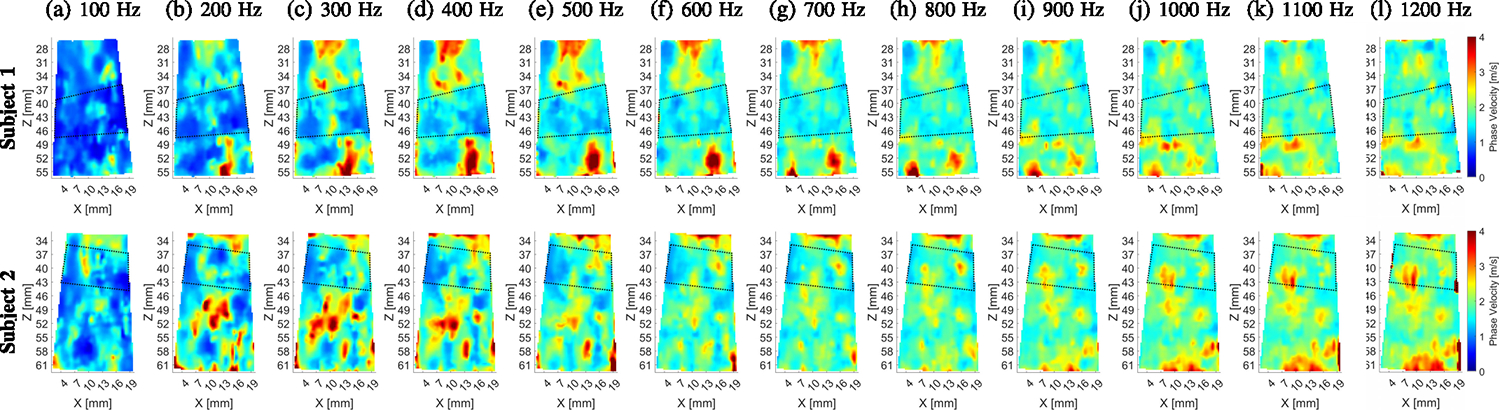
2D shear wave phase velocity images reconstructed in the kidney cortex using the USEWEB method for selected frequencies from 100 Hz to 1200 Hz with an interval of 100 Hz are shown in figures (a)-(l), respectively. The images were calculated for a constant spatial window dimension of 3.10 × 3.10 mm. Dashed lines present manually selected locations of the cortex estimated from B-mode images in Fig. 12.

**Fig. 14.**
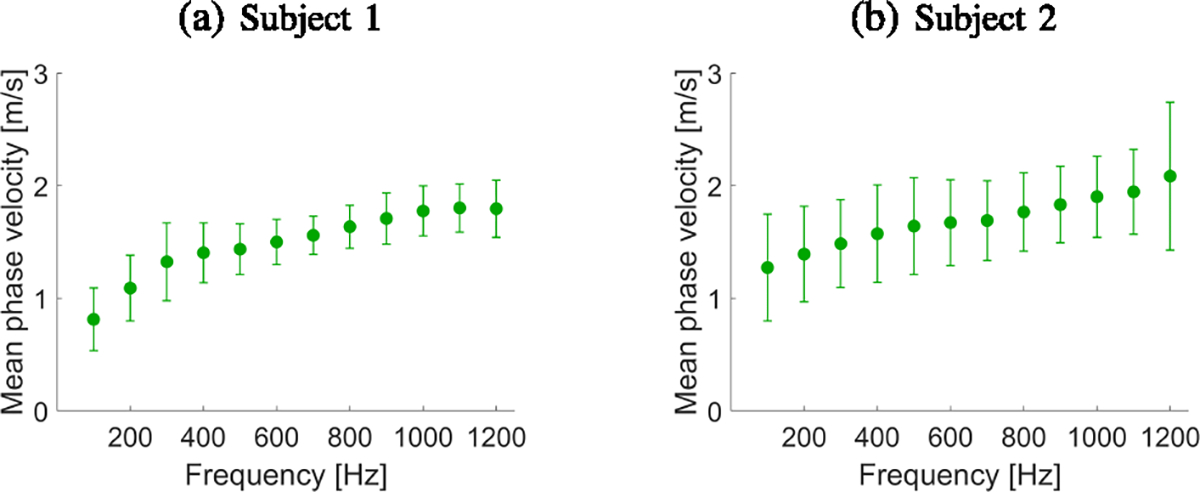
Dispersion phase velocity curves calculated for the *in vivo* renal transplants using the mean and standard deviation of the phase velocity for USEWEB presented in Fig. 13. The results were calculated for selected ROIs (renal cortex) presented in Fig. 13c.

Figure 12 shows B-mode images of longitudinal profiles of the kidneys with the marked area where the measurements were made. Figure 13 displays reconstructed 2D phase velocity images for two *in vivo* renal transplants obtained using the USEWEB approach. The images were calculated for a constant spatial window dimension of 3.10 × 3.10 mm. The phase velocity maps with texture are distinguishable, and an increase in shear wave velocity with frequency can also be observed. Figure 14 shows dispersion phase velocity curves calculated from reconstructed images for manually selected ROI from the renal cortex based on B-mode images. An increase in phase velocity with frequency is present. This phenomenon likely arises due to the viscoelastic nature of the kidneys.

## V. DISCUSSION

This study presents a novel method called USEWEB for imaging shear wave velocity in soft tissues over a wide bandwidth. The technique operates in the frequency domain, similar to LPVI, and generates images for specific frequencies. The study investigates three types of materials: homogeneous elastic, homogeneous viscoelastic, and heterogeneous elastic materials, with all data being experimental measurements that contain realistic acquisition noise.

The USEWEB method utilizes the S-transform, which combines the strengths of the short time Fourier transform (STFT) and the continuous wavelet transform (CWT) methods, while overcoming their limitations. The STFT is limited to single-resolution analysis and produces spectral smearing due to windowing. Additionally, due to the fixed window width, it cannot accurately track the dynamics of the signal. The CWT is a multi-resolution method; however, it results in a time-scale decomposition instead of a time-frequency decomposition, and its temporal resolution is frequency-dependent and controlled by the analyzing wavelets’ range [25]. On the other hand, the S-transform is a multi-resolution method that defines the S-transform of a function h(t) as a CWT with a specific mother wavelet multiplied by a phase factor of $e^{i2\pi f\tau }$. It extends the instantaneous frequency to broadband signals. The S-transform phase referenced to the time origin offers supplementary insights into spectra that cannot be obtained from locally referenced phase information in the CWT. It includes phase factors that solely pertain to the local phase information of each signal component. The USEWEB method utilizes the amplitude and phase spectrum of the S-transform to estimate the phase velocity of the shear wave.

The upper frequency for the homogeneous phantoms and the *in vivo* data was restricted by the Nyquist frequency for the motion detection process. Tissue-mimicking phantoms were scanned using a programmable ultrasound research system with an effective frame rate of 4167 Hz, while in the case of *in vivo* renal transplant data acquisition, a General Electric Logiq E9 ultrasound system was used, and shear wave motion was measured with ultrasound data at a frame rate of 2410 Hz. For the mentioned sampling rates, based on the Nyquist frequency, the upper frequency limits are 2083 Hz, and 1205 Hz, respectively.

Most soft tissues are viscoelastic, which imposes higher attenuation resulting in reduced propagation distance compared to elastic materials. Shear wave velocity is frequency-dependent due to tissue viscosity, making viscoelastic materials difficult to image compared to elastic materials. One approach to mitigate this challenge is to use a bandpass filter in the wavenumber domain (k-filter), centered around the nominal wavenumber of the shear wave-mode, estimated beforehand from the dispersion curve [22]. However, this may not always be the optimal approach for certain applications. While the k-filter improves image reconstructions for certain frequencies, it does not increase the frequency bandwidth for higher frequencies (Figs. 3 and 6). This study introduces a new 2D shear wave elastography method in the frequency domain, which does not require bandpass filters in the wavenumber domain to obtain accurate and robust results.

The study demonstrates the effectiveness of USEWEB in producing 2D shear wave phase velocity maps with low variability over a wide frequency range, indicating promising results for its potential use in various clinical applications. As demonstrated in this study, the proposed technique is capable of reconstructing shear velocity images for materials with diverse mechanical properties ranging from soft (E = 10 kPa, Fig. 2 top row) to stiff (E = 45 kPa, Fig. 2 bottom row), and with varying viscosity from low (Phantom A, Fig. 5 top row) to high (Phantom C, Fig. 5 bottom row). Furthermore, the usefulness of the proposed technique was demonstrated for imaging heterogeneous materials using a CIRS phantom with cylindrical inclusions and using *in vivo* renal transplants.

The reconstructions from experimental data in this study have demonstrated that using the USEWEB technique for both elastic and viscoelastic materials results in improved phase velocity maps compared to the LPVI approach. The USEWEB method is able to overcome the issues encountered by the LPVI approach at higher frequencies for elastic phantoms and over a wide frequency range for tissue-mimicking viscoelastic phantoms. Consequently, the reconstructed phase velocity maps align well with the dispersion phase velocity curves calculated for the focal depth with much lower coefficient of variation (CV < 5%), as depicted in Figs. 3, 4, 6, and 7.

The inclusion phantom experiments in this study revealed that the USEWEB technique is also capable of providing more reliable contrast between the inclusion and the surrounding background, compared to the LPVI method. The results also suggest that USEWEB is suitable for imaging small inclusions with sizes smaller than 5 mm with accurate wave velocities acquired at higher frequencies, >800 Hz. The cylindrical design of the inclusions in the CIRS phantom implies that the image cross-sections should be circular, and the reconstructed inclusions were indeed circular for the dimensions investigated at higher frequencies, as seen in Figs. 8, and 11.

Similar to the LPVI method, the spatial resolution of the USEWEB technique is determined by the spatial wavelengths, which become shorter at higher frequencies, and the size of scanning window dimension, shown in Fig. 9. Ideally, one or more wavelengths are required to achieve accurate estimation of the shear wave velocity. At the same time, these wavelengths should be in regions where the local spatial window fully covers inclusion and does not overlap with background material. This is crucial for effectively resolving small inclusions with high contrast and accurate values.

The shape distortions in heterogeneous materials, in general, depend on both the window size and frequency. The shape distortions are influenced by frequency because the spatial resolution of the USEWEB and LPVI methods is determined by the spatial wavelengths, which are shorter at higher frequencies (λ=c/f). The shear wave wavelength decreases as frequency increases, resulting in a reduced transition region and a reduction in underestimation bias for the inclusion (material interface).

Frequencies ranging from 400 to 1800 Hz were utilized in a heterogeneous CIRS phantom in Figs. 8, 10 and 11. This frequency range corresponds to spatial wavelengths ranging from 12.9 mm to 2.87 mm, respectively, for the Type IV inclusion with a nominal wave speed of 5.16 m/s. Therefore, this range was deemed the most suitable for investigation as it covers a wide enough range of wavelengths compared to the inclusion size. Specifically, at 12.9 mm, the wavelength is twice the size of the inclusion, while at 2.87 mm, the wavelength is more than two times smaller than the inclusion size.

The USEWEB algorithm assumes local homogeneity of the material within a spatial window size, which means that the material properties within the window are assumed to be uniform. However, this assumption is often violated near boundaries between regions with different stiffness. When there is a boundary between two regions with different stiffness, the shear wave velocity gradually transitions between the two regions. This means that near the boundary, the shear wave velocity is elevated in the background region and diminished in the inclusion region. The local homogeneity assumption of the USEWEB algorithm does not account for this gradual transition, and as a result, the estimated shear wave velocity profile may be inaccurate near these boundaries, as shown in Fig. 9. To address this issue, several modifications could be added to the USEWEB algorithm, which are out of the scope of this work. One approach could be to incorporate a transition zone model that accounts for the gradual change in shear wave velocity near boundaries. These modifications could help to improve the accuracy of the estimated shear wave velocity profile near boundaries between regions with different stiffness.

For both techniques, the USEWEB approach and the LPVI method, using a smaller spatial window size can improve the spatial resolution of shear wave velocity estimates, but choosing a window that is too small can result in a noisier image with blurring. Therefore, the optimal spatial window size should depend on the size of the inclusion being evaluated.

The CNR of the USEWEB method surpasses that of the LPVI approach. It has been demonstrated that as the frequency increases, the CNR of the USEWEB method also increases and remains above 23 dB. The CNR of LPVI, on the other hand, begins to decrease at 1000 Hz for the inclusion size of 6.49 mm, and at 600 Hz for the inclusion size of 4.05 mm. It is worth noting that the CNR will change if a different spatial window size is used.

Two exemplary *in vivo* renal transplants were used to demonstrate the feasibility of the USEWEB approach used in clinical applications. The USEWEB approach allows for the local analysis of phase velocity dispersion by reconstructing images from which specific ROIs can be selected and further analyzed if needed. Having the ability to explore tissue wave velocities at higher frequencies that were previously not measurable could open up the ability for more advanced viscoelastic characterizations [33].

The data acquired using the GE Logiq E9 was from an ongoing study. The Verasonics would provide more flexibility for *in vivo* experiments, but the aim of the study was to use hardware that would have direct translation as opposed to the Verasonics, which is only a research platform. The selection of the ROI size by practitioners can be influenced by their understanding of the f# employed during the application of ARF pushes. Furthermore, the maximum allowable ROI size might be predetermined by the manufacturer in alignment with the ARF f#. Determining the optimal window size can be based on various metrics, potentially involving user interaction. These metrics might include measures of contrast between two regions or reducing the coefficient of variation (the ratio of standard deviation to the mean) or the interquartile to median ratio. Users can define regions or inclusions, and the window size can be optimized to meet a user-defined threshold, balancing the need to preserve resolution with smaller windows while reducing image variation. A window size can also be predetermined for investigating both homogeneous and heterogeneous materials. For example, one could choose a window size of 4.0×4.0 mm based on these findings, ensuring that the CV coefficient is below 5% for homogeneous materials and that the CNR exceeds 25 dB in heterogeneous scenarios. Based on the phase velocities shown in Figs. 3 and 6, we can deduce the frequency range in which practitioners can depend on precalculated 1D dispersion curves at the focal depth utilizing the GST-SFK method.

The main advantages of the USEWEB method and its novelty can be summarized as follows. The USEWEB method offers the unique capability of constructing 2D images for viscoelastic phantoms/tissues across an extended frequency range, surpassing the capabilities of other known methods. This extended frequency range enables enhanced differentiation of the local viscoelastic behavior of the tissue, as demonstrated in the study by Kijanka et al. [26]. The authors conducted a thorough examination of the behavior of viscoelastic parameters as the frequency range expanded. The analysis focused on observing if these parameters reached a plateau and maintained consistent values, indicating convergence of the Zener and Kelvin-Voigt models to the data sets under investigation. At higher frequencies, the distinction between the viscoelastic materials becomes more prominent, providing greater separation compared to lower frequencies. A comprehensive comparison between the USEWEB and LPVI approaches demonstrates that the USEWEB method provides robust results with low coefficients of variation. Moreover, the USEWEB method exhibits heightened reliability when utilizing a smaller spatial window size in contrast to the LPVI approach, leading to a significant reduction in the coefficient of variation. This is very important in the context of imaging of cancerous lesions and providing accurate results with high resolution and contrast. An additional advantage of the USEWEB method is that it does not require a bandpass filter in the wavenumber domain, thereby preventing the potential loss of crucial information that may occur with such filtering.

The primary drawback of this method is its computationally intensive reconstruction process, which increases with the window size and the number of frequencies utilized. To overcome this limitation, future research will explore ways to optimize the method. Another limitation of this work is the primary use of phantoms. Future work will be directed towards evaluation the robustness of the method for *in vivo* imaging, however preliminary feasibility was demonstrated with data from a clinical ultrasound scanner from *in vivo* acquisitions.

## VI. CONCLUSION

This paper presents a new technique called USEWEB for imaging shear wave velocity and demonstrates its effectiveness in generating 2D phase velocity maps across a wide frequency range. The paper shows that USEWEB can accurately reconstruct 2D phase velocity maps in homogeneous elastic and viscoelastic phantoms with various viscoelastic parameters with CV < 5%, and also provides good contrast between inclusions and the surrounding background with limited artifacts. More importantly, the USEWEB method does not require the use of a narrow band-pass filter in the wavenumber domain to limit the shear wave modes in the processed data. Future work includes conducting additional *in vitro* and *in vivo* experiments on viscoelastic tissues to further explore the potential of USEWEB.

## Figures and Tables

**Fig. 15.**
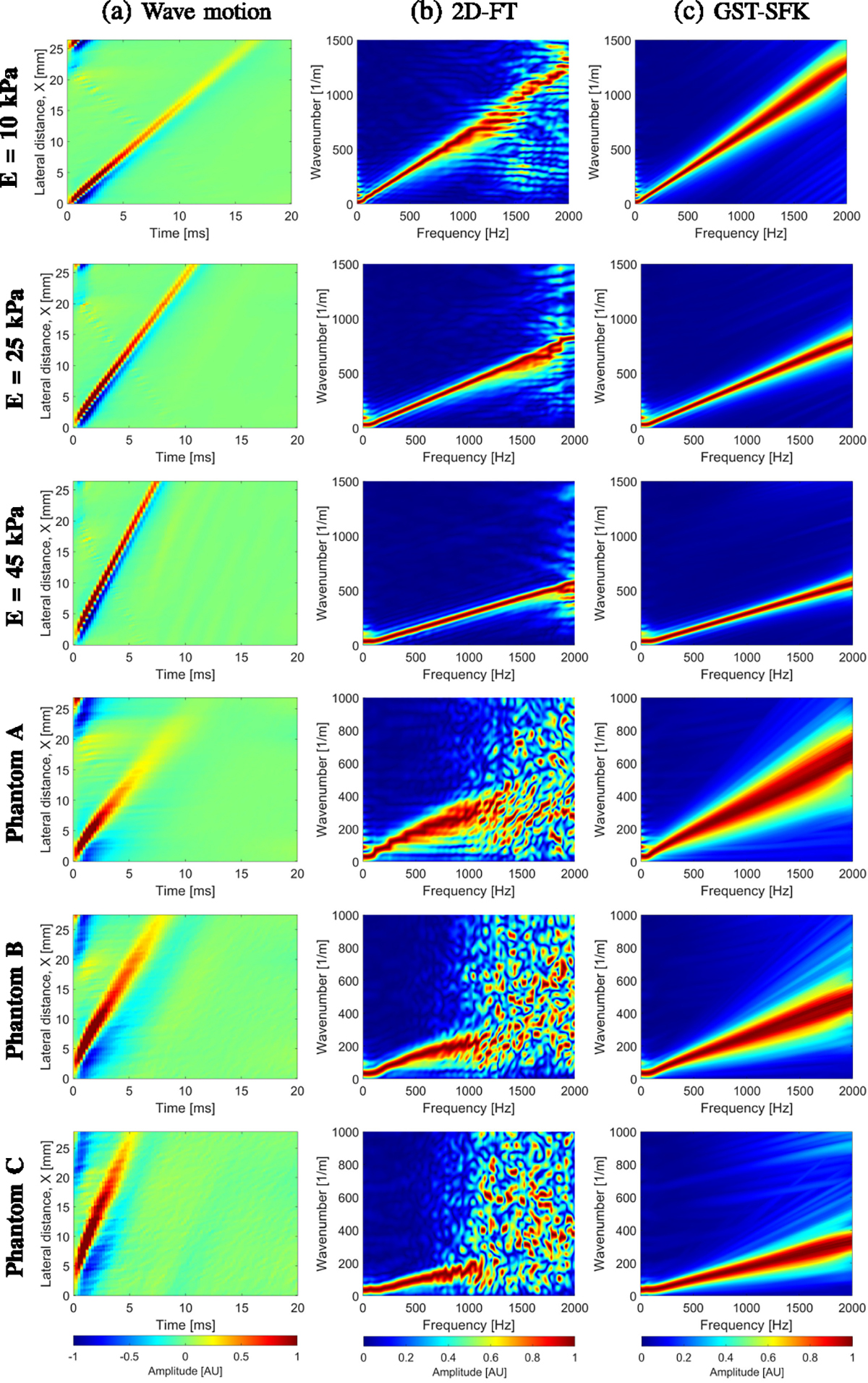
(a) Shear wave velocity motion data. The frequency-wavenumber (f-k) distribution reconstructed based on the (b) 2D-FT, and (c) GST-SFK methods. The f-k maps are normalized by wavenumber maxima in the frequency direction individually for each frequency to show the data optimally. Results were calculated for the experimental, custom-made TM elastic phantoms with E = 10, 25, and 45 kPa, and viscoelastic phantoms A, B, and C.

## APPENDIX

Figure 15 shows the frequency-wavenumber (f-k) distribution reconstructed based on the (b) 2D-FT, and (c) GST-SFK methods, for shear wave particle velocity motion measurements in (a). The f-k maps are normalized by wavenumber maxima in the frequency direction. Results were calculated for the experimental, custom-made TM elastic phantoms with E = 10, 25, and 45 kPa, and viscoelastic phantoms A, B, and C.

Differences are apparent when comparing the results obtained with the 2D-FT and GST-SFK methods. Specifically, for E = 10 kPa, 25 kPa, and 45 kPa, frequencies exceeding around 1000 Hz, 1500 Hz, and 1800 Hz, respectively, show predominantly noisy f-k maps when using the 2D-FT approach. These variances are significantly more noticeable when dealing with viscoelastic phantoms. In the case of the 2D-FT approach, noise dominates the results for frequencies above approximately 900 Hz. However, the GST-SFK technique has been effective in minimizing the impact of noise.
